# Intermediate neoadjuvant radiotherapy for T3 low/middle rectal cancer: postoperative outcomes of a non-controlled clinical trial

**DOI:** 10.18632/oncotarget.2603

**Published:** 2014-10-18

**Authors:** Giovanni Bisceglia, Nicola Mastrodonato, Berardino Tardio, Gianluigi Mazzoccoli, Pietro Corsa, Michele Troiano, Salvatore Parisi

**Affiliations:** ^1^ Department of Surgical Sciences, Division of Abdominal Surgery, IRCCS Scientific Institute and Regional General Hospital “Casa Sollievo della Sofferenza” San Giovanni Rotondo, Italy; ^2^ Department of Medical Sciences, Division of Internal Medicine and Chronobiology Unit, IRCCS Scientific Institute and Regional General Hospital “Casa Sollievo della Sofferenza” San Giovanni Rotondo, Italy; ^3^ Department of Radiological Sciences, Division of Radiotherapy, IRCCS Scientific Institute and Regional General Hospital “Casa Sollievo della Sofferenza” San Giovanni Rotondo, Italy

**Keywords:** T3 rectal cancer, preoperative radiotherapy, local control, survival, local recurrence, distal recurrence

## Abstract

**Background:**

The benefits of adjuvant radiotherapy in rectal carcinoma are well known. However, there is still considerable uncertainty about the optimal radiation treatment. There is an ongoing debate about the choice between very short treatments immediately followed by surgical resection and prolonged treatments with delayed surgery. In this paper, we describe an interim analysis of a non-controlled clinical trial in which radiotherapy delivered with intermediate dose/duration was followed by surgery after about 2 weeks to improve local control and survival after curative radiosurgery for cT3 low/middle rectal cancer.

**Methods:**

Preoperative radiotherapy (36 Gy in 3 weeks) was delivered in 248 consecutive patients with cT3NxM0 rectal adenocarcinoma within 10 cm from the anal verge, followed by surgery within the third week after treatment completion.

**Results:**

166 patients (66.94%) underwent anterior resection, 80 patients (32.26%) the Miles' procedure and 2 patients (0.8%) the Hartmann's procedure. Local resectability rate was 99.6%, with 226 curative-intent resections. The overall rate of complications was 27.4%. 5-year oncologic outcomes were evaluated on 223 patients. The median follow-up time was 8.9 years (range 5-17.4 years); local recurrence (LR) rate and distal recurrence (DR) rate after 5 years were 6.28% and 21.97%, respectively. Overall survival was 74.2%; disease free survival was 73.5%; local control was 93.4 % and metastasis-free survival was 82.1%.

**Conclusions:**

preoperative radiotherapy with intermediate dose/duration and interval between radiotherapy and surgery achieves high local control in patients with cT3NxM0 rectal cancer, and high DR rate seems to be the major limitation to improved survival.

## INTRODUCTION

Over the past few decades, significant advances in the treatment of rectal cancer have been made. The introduction of high-dose radiotherapy (RT) in the preoperatory period has led to a 50% reduction in local recurrence (LR) rate [1-5]. Subsequently, the introduction of the total mesorectal excision (TME) into surgical practice has allowed further reduction in LR rate below 10% [6,7]. The role of adjuvant therapies, either with or without fluorouracil-based chemotherapy (CHT), remains controversial in terms of improvement of overall survival [8-10], with a possible risk of overtreatment in patients with T3 rectal cancer. Our study started in 1988 on the basis of the French [1] and Swedish works [2] on radiotherapy, with the aim of improving outcomes in rectal cancer. RT was planned in the preoperative time and was delivered with midway dose/duration and interval between radiotherapy and surgery. The Uppsala trial showed that preoperative RT was significantly better tolerated and gave a significantly better local control than postoperative radiotherapy[2]. RT protocols are different from standard regimens. In our study the planned treatment (36 Gy in 3 weeks) was characterized by an “intermediate fraction”, and was similar to the one used in the Lyon R90-01 trial, where a total dose of 39 Gy was delivered (3.0 Gy per fraction in 13 fractions within 2 weeks) [12]. By applying a 2-week break between RT and surgery, which could be named “intermediate interval”, it is possible to take advantage of both the Swedish short interval cell-killing effect and the conventional long interval downstaging/sizing effect. Results of trials comparing local recurrence rate in preoperative RT and in surgery alone, - EORTC-40761(LR = 15% vs. 30%; P = .003) [1], Stockholm I (LR = 13% vs. 30%; P = .001) [3], SRCG (LR = 9% vs. 26%; P = .001) [5] - showed that preoperative RT reduces local recurrence rates among patients with resectable rectal cancer by more than 50%. In 1992, on the basis of the Heald's study [13], we introduced the total mesorectal excision (TME) into our surgical practice as this surgical technique was found to markedly reduce LR. In addition, the Dutch Colorectal Cancer Group Trial demonstrated that preoperative RT maintains its benefit and, when combined with TME, reduces LR rate below 10% (LR = 5% vs. 11%; P = .001) [14]. These data supported our experience, in fact we were able to achieve high local control in patients with local advanced rectal cancer, as reported in our previous studies, where we showed a 5-year LR rate of approximately 6% in patients with cT3NxM0 clinical stage [15, 16].

In this study, we report on a radiosurgery protocol in which RT was delivered with intermediate dose/duration and was followed by surgery after about 2 weeks. We describe the postoperative outcomes of a prospective mono-institutional study on patients with cT3NxM0 rectal cancer whose median follow-up was 8.9 years. We show the results of local control obtained by using this radiosurgery treatment, which is middle between the traditional ones; we used this protocol with the aim of reducing the incidence of LR, and we considered necessary to select all those patients who had been observed for at least 5 years. Moreover, we report what happened after these 5 years: in our experience, we have not registered LR, suggesting that the chosen treatment has a number of potential advantages, both with respect to the “short” 5 days treatment and compared to the “long” 5 weeks treatment.

## RESULTS

### Patients' Characteristics

This study was planned for local advanced rectal cancer (cT3/cT4 NxM0) in January 1988, and 319 consecutive patients were enrolled until 2013. From January 1988 to December 2008, 248 consecutive cT3 rectal cancer patients were selected. One hundred sixty-seven (67.34%) were men and 81 (32.66%) were women; the median age at surgery was 65 years (SD 9.8; range 28 - 81 years). One hundred thirty-eight tumors were located in the lower third of the rectum and 110 in the middle third of the rectum. The median tumor level was 5 cm from the anal verge (SD 2.4; range 2 - 10 cm). Patients' and treatment's characteristics are shown in Table 1.

**Table 1:** Patients' Characteristics

	All patients			Curative resection		Palliative resection	
	N	%		N	%	N	%
	248	100		226	91.13	22	8.87
Sex							
Male	167	67.34		152	67.26	15	68.18
Female	81	32.66		74	32.74	7	31.82
Age (years)							
Range	27 - 81			30 - 81		27 - 77	
Median	65			65		63	
Tumor level (cm)							
Range	2 - 10			2 - 10		2 - 10	
Median	5			5		6	
Type of surgery							
Miles's procedure	80	32.26		75	33.19	5	22.73
Anterior resection	166	66.94		151	66.81	15	68.18
Hartmann's procedure	2	0.80				2	9.09
Residual disease	1	0.4			-	1	4.55
pT pN pM							
pT0	6	2.42		6	2.65		
pTis	2	0.81		2	0.88		
pT1	3	1.21		3	1.33		
pT2	50	20.16		47	20.80	3	13.64
pT3	181	72.98		164	72.57	17	77.27
pT4	6	2.42		4	1.77	2	9.09
pN0	164	66.13		158	6.91	6	27.27
pN1	62	25.00		51	22.57	11	50.00
pN2	22	8.87		17	7.52	5	22.73
pM	21	8.47				21	95.45

### Preoperative Radiotherapy

All 248 patients well tolerated RT; possible acute side effects, such as diarrhea, tenesmus, dysuria, were of short duration and were successfully treated with the symptomatic medical care. None of the patients interrupted the treatment. The median RT duration was 16 days (range 15 – 17 days). The 2-week planned interval before surgery had a median duration of 19.0 days (SD 5.5; range 9 – 47 days). The overall median duration of the radiosurgical treatment was 35 days (SD 5.5; range 24 – 63 days).

### Surgery

Two hundred twenty-six patients underwent curative-intent surgery: 151 patients (66.81%) underwent sphincter saving (SS) surgery, and 75 (33.19%) underwent sphincter demolition surgery (DS). Twenty-one patients (8.47%) had distant metastasis at surgery, which was undetectable preoperatively, and 1 patient (0.4%) underwent non-radical surgery due to residual disease. The local resectability rate was 99.6% (Table 1).

### Postoperative Mortality and Morbidity

Postoperative mortality and morbidity rates were calculated for all patients. Sixty-eight patients (27.4%) had postoperative complications with a mortality rate of 2.02% (5 out of 248 patients). The specific morbidity rate associated with radiosurgery was 16.1% in SS vs. 36.3% in DS (P= 0.0004); whereas the overall complications significantly increased in the DS group (20.8% SS vs. 41.3% DS; P=0.0001). No statistically significant differences in the general morbidity and mortality rate were observed (Table 2).

**Table 2:** Post-operative mortality and morbidity

	*Patients*		*Surgery*				*P*
			SS		DS		
	248		168		80		
	n°	%	n°	%	n°	%	
*General morbidity*							
Renal Failure	1	(0.4)			1	(1.2)	
Cerebral ischemia	2	(0.8)			2	(2.5)	
Pulmonary embolism	1	(0.4)	1	(0.6)			
Ascitic decompesation	1	(0.4)	1	(0.6)			
Acute iliac trombosis	1	(0.4)	1	(0.6)			
Pleural effusion	1	(0.4)	1	(0.6)			
	7	(2.8)	4	(2.4)	3	(3.7)	ns
*Specific morbidity (related to radio-surgical treatment)*							
							
Intra-abdominal bledding	1	(0.4)	1	(0.6)			
Intra-abdominal infection	2	(0.8)	2	(1.2)			
Ureteral necrosis	1	(0.4)			1	(1.2)	
Bladder failure	11	(4.4)	11	(6.5)			
Abdomen wound: suppuration	2	(0.8)			2	(2.5)	
Perineal cavity: suppuration	21	(8.5)			21	(26.3)	
Perineal cavity: hemorrhage	2	(0.8)			2	(2.5)	
Fistula	2	(0.8)			2	(2.5)	
Ureteral lesion	1	(0.4)			1	(1.2)	
Anastomotic dehiscence	13	(5.2)	13	(7.7)			
	56	(22.6)	27	(16.1)	29	(36.3)	.0004
*Total morbidity*	63	(25.4)	31	(18.5)	32	(40.0)	.0003
*Mortality*	5	(2.0)	4	(2.4)	1	(1.3)	ns
	68	27.4	35	(20.8)	33	(41.3)	.0008

SS: sphincter saving; SD: sphincter demolition. Values in parenthesis are percentages

### Pathologic Characteristics

The comparative assessment of clinical and pathological T staging revealed a pathological complete remission (pCR) in 6 patients (2%); instead, upstaging was found in 6 patients (2%) and downstaging in about 25% of cases (Table 1). The details of the pathologic characteristics are listed in Table 3.

**Table 3:** TNM anatomical and pathological staging (patients with ≥ 5-year follow-up)

*TNM Stage*				*Patients*	%
0		T0	NO	6	
		T in situ	N0	2	
				8	3.6
I		T1	N0	3	
		T2	N0	40	
				43	19.3
II		T3	N0	103	
		T4	N0	1	
				104	46.6
III		T2	N1	5	
		T2	N2	1	
		T3	N1	43	
		T3	N2	16	
		T4	N1	3	
				68	30.5
	Total			223	100

### Follow-up

Twenty-one out of 248 eligible patients who had liver metastasis at surgery were excluded from the study; 1 patient was resection margin positive and 3 patients died due to postoperative complications. In total, 223 patients were eligible for follow-up. The median follow-up time of alive patients was 8.9 years (range 5 to 17.4 years).

### Local recurrence

The 5-year LR rate was 6.28% (14 out of 223 patients) and was observed at a median of 25.5 months (SD 12.3; range 11.4 – 53 months). Both LR and DR were observed in 7/14 patients (50%). Five patients had stage II disease and 9 had stage III. No downstaging was detected: 13 patients had stage ypT3 and 1 patient had stage ypT4 (upstaging). No statistically significant differences were found for sex, tumor level (cutoff 5 cm), TME, or type of surgery. No local recurrences were found after the 5^th^ year from surgery (Table 4).

**Table 4:** Disease progression after ≥ 5- year follow-up

	N	%
Local recurrence	14/223	6.28
Distant recurrence	49/223	21.97

### Distant recurrence

The 5-year DR rate was 21.97% (49 out of 223 patients) and was observed at a median of 17 months (SD 12.0; range 3 – 50 months). Four patients had stage I disease, 25 had stage II and 20 had stage III. The incidence of DR was not influenced by sex or type of surgery. After the 5^th^ year from surgery, distant metastases were found in 2 patients. (Table 4).

### Survival

**Figure 1:**
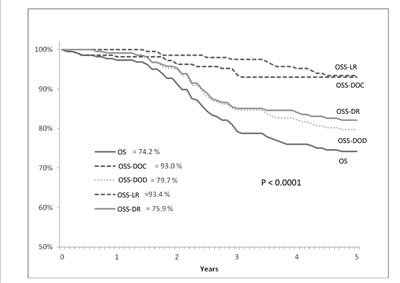
Cumulative survival (y axis) and overall survival (x axis) evaluated according to Kaplan-Meier statistical analysis and disease status in T3 low/middle rectal cancer patients treated witheoadjuvant radiotherapy and with ≥ 5-year follow-up OS = Overall survival, DR = Distal recurrence, LR = Local recurrence, OSS-DR = Overall specific survival-DR, OSS-LR = Overall specific survival-LR, DOC = Patients who died with no evidence of disease, DOD = Patients who died with evidence of disease (local or distal).

**Figure 2:**
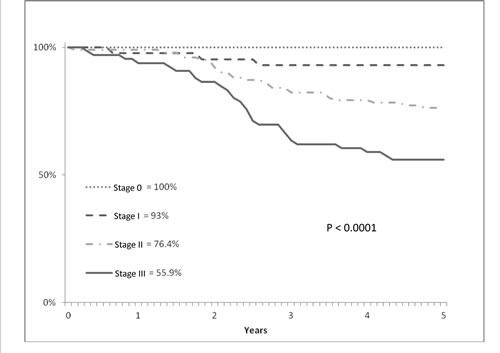
Cumulative survival (y axis) and overall survival (x axis) evaluated according to Kaplan-Meier statistical analysis and pathological stage in T3 low/middle rectal cancer patients treated witheoadjuvant radiotherapy and with ≥ 5-year follow-up

Table 5 shows the patients' status after 5 years from surgery; the number of alive patients was 159 (71.3%). Local and/or distant recurrence was observed in 63 patients (28.25%), contributing to the 5-year disease free survival (DFS) rates, which were estimated at 73.5% (SE 3.0). The 5-year cancer-specific survival rate was 79.7% (SE 2.8), with a LR-specific survival rate of 93.4% (SE 1.8) and a DR-specific survival rate of 82.1% (st.er.: 2.7). The 5-year overall survival (OS) rate was 74.2% (SE 3.0) (Table 6; Figure 1). No significant differences for sex, tumor level and type of surgery were found in OS; but significant differences in the stage distribution were registered (Figure 2).

**Table 5:** Status after ≥ 5-year follow-up

	All patients			Palliative			≥ 5-year follow-up	
	N	%		N	%		N	%
								
No evidence of disease	146	58.87			-		146	65.47
Alive with disease	16	6.45		3	13.64		13	5.83
Death from disease	60	24.19		16	72.72		42	18.83
Death from other cause	15	6.05			-		14	6.28
Lost to follow-up	11	4.44		3	13.64		8	3.59
	248	100		22	100		223	100

**Table 6:** Survival after ≥ 5-year follow-up

SURVIVAL		Estimate %	Standard Error
*Overall survival (OS)*		74.2	3.0
*Overall specific survival (OSS)*			
	DOC	96.3	1.3
Cancer specific survival	DOD	79.7	2.8
	LR	93.4	1.8
	DR	82.1	2.7
			
Disease free survival (DFS)		73.5	3.0
			
Disease free specific survival (DFSS)	LR	92.2	2.0
	DR	76.6	2.9

DOC: death from other cause; DOD: death from the disease; LR: local recurrence; DR: distant recurrence

### Protocol violations

The protocol was violated in two 81-year old patients.

## DISCUSSION

The advantages of adjuvant RT in rectal carcinomas are recognized, but up till now there is significant uncertainty regarding the most favorable radiation treatment. In particular, there is still a long-lasting dispute concerning the option between very short treatments (25 Gy in 5 days) immediately followed by surgical resection and prolonged treatment (45-50 Gy in 5 weeks) with delayed surgery. Both choices have their distinct advantages and disadvantages. We opted for an innovative experience and an intermediate choice was made in which a slightly accelerated RT (36 Gy in 3 weeks) was followed by surgery after an interval of about 2 weeks. The interesting aspect of this experience is that this treatment modality allows to obtain a compromise between the two RT protocols today considered as standard. In particular, the treatment in 3 weeks could favor downstaging of the disease (compared to “short” treatment) maintaining a reasonable duration of the integrated radio-surgical treatment (compared to “long” treatment). The long duration of follow-up and the large number of cases contribute to enrich our study, anyway we could argue that our clinical staging was “sub-optimal” for two reasons. First of all, we reported a 25% downstaging rate, but then, as we also had 6 ypT4 patients, we had to consider a lack in the real rate of cT4. The second point is that our department did not gain any advantage from the magnetic resonance imaging (MRI) because pelvic MRI was introduced into our clinical practice only few years ago. Nowadays, MRI is considered the gold standard in rectal cancer imaging since it achieves a clinical “T” staging which is very close to pathological staging. MRI can accurately detect the mesorectal fascia, is able to assess the invasion of the mesorectum or of the surrounding organs, and effectively predicts the circumferential resection margin, even if nodal disease remains a difficult radiological diagnosis [17]. For these reasons, MRI plays a pivotal role in the patients' selection process, but it also has a prognostic role as it is fundamental in the choice of the appropriate therapeutic strategy. Also the results from the MERCURY trial showed that preoperative MRI could define the group of stage II and III disease patients with a good prognosis, who were likely to be treated with surgery alone. A 5-year LR of 3% in the total population and of only 1.7% in MRI good prognosis T3 stage patients were achieved^.^[18]. A Mayo Clinic retrospective review of patients with rectal cancer treated with curative-intent surgery alone showed a 5-year rate of LR of 4.3% [19]. The additional therapy adds morbidity to that caused by surgery, and should therefore be administered only when the risk of LR is sufficiently high [10]. Among all the trials comparing surgery alone with RT, increased postoperative mortality has been observed only in the Stockholm I trial, in which large fields of RT were adopted [3]. The Polish trial showed that the rate of all complications in patients treated with the 5×5 Gy schedule was 24% vs. 85% in patients who received preoperative chemoradiotherapy (CRT) (P= .0001); furthermore, grade III and IV (including death) complications among patients treated were 3 vs. 18%, (P= .0001), respectively [20]. In the FFCD 9203 trial, the overall rate of grade III to IV toxicities, according to the WHO scale, was significantly higher in the CRT arm (14.9%) than in the RT arm (2.9%; P= .0001) [21]. In the EORTC 22921 trial, grade II acute toxic effects were reported in 38.4% of patients receiving preoperative CRT, and grade III or higher acute toxic effects occurred in 7.4% of cases [22]. Also, it is worth noticing that 20% of patients of both studies did not receive the 5-fluorouracil planned dose. In our experience, RT caused an increase in the total time of treatment, with a median of 35 days. All patients completed the radiosurgical protocol (compliance 100% of patients). The overall complications rate (including deaths) was 27.4%. The real effect of adjuvant therapies on the improvement in overall survival is still debatable. The Swedish Rectal Cancer trial showed and confirmed that preoperative RT improves survival [5]; this can be explained by the “marked” reduction in the risk of LR after RT; in contrast, the rate of distant metastases was not influenced [23]. Also the Stockholm II trial reported that LR rate reduction and improved survival were obtained in the patients who underwent curative-intent surgery [4]. This effect cannot be verified in terms of OS in TME trials, where the improvement in local control achieved with TME decreases the “marked” reduction obtained by RT, which does not translate into an improved overall survival. The results of a randomized trial comparing preoperative RT alone vs. neoadjuvant CRT, such as the Polish trial or the FFCD 9203 trial, - with or without TME – concluded that there was no impact on overall survival for stage T3 or T4 resectable rectal cancer [20,21]. The Australian intergroup trial showed no difference on OS for clinically staged T3 cancer[24]. The EORTC 22921 trial concluded that in patients with stage T3 or T4 resectable rectal cancer treated with preoperative RT the association of CHT preoperatively or postoperatively produced no significant effect on survival[22,25]. Historically, postoperative CRT has proved to effectively reduce local recurrences and to improve survival for locally advanced rectal cancer [26, 27]. Between 1993 and 1994, prospective randomized trials aimed at comparing the efficacy of preoperative with postoperative CRT were started. Two trials were performed in the United States – the Radiation Therapy Oncology Group (RTOG) 94-01 trial and the National Surgical Adjuvant Breast and Bowel Project (NSABP) R-03 trial-; another trial was started by the German Rectal Cancer Study Group (the CAO/ARO/AIO-94 trial) [28]. Unfortunately, the RTOG 94-01 trial enrolled only 53 patients and was closed prematurely. Also the NSABP R-03 trial failed to reach the planned goal of 900 patients as it enrolled only 267 patients between 1993 and 1999. This trial showed a significantly improved disease-free survival in the preoperative CRT arm but no improvement in OS and local control [29]. Moreover, after a median follow-up of 11 years, the CAO/ARO/AIO-94 trial concluded that no effect on OS was obtained [30]. In all the analyzed studies, there are many different opinions on the effects of adjuvant therapies on survival, while the authors agree on the fact that adjuvant therapy has no effect on DR. It seems that adjuvant therapies affecting LR may be able to reduce a part of the features that make rectal cancer a dismal nosological entity; the fact that adjuvant therapies have no effect on DR makes colon and rectal cancer similarly grim. Only systemic treatments which are able to affect DR will be able to have an effect on cancer specific survival and consequently on OS. In our report, the 5-year rate of DR was 22.97%. Indeed, we found that cancer specific survival can be more influenced by DR, with specific survival about 82.1%, respect to LR, with specific survival about 93.4%. Another advantage of CRT could be a better anal-sphincter preservation[31,32]. However, there is no firm evidence supporting this hypothesis [20-22]. The goal of improved sphincter preservation by neoadjuvant treatment remains complex and surgeon-dependent. The final decision on sphincter preservation is not based at the time of surgery, but on tumor status before irradiation. In the Lyon R90-01 trial, as much as 9% LR rate was found, and in patients who underwent sphincter preservation, where conservative surgery did not seem possible at the beginning, LR occurred in 12% of cases [33]. It is the surgeon's responsibility to choose between abdominoperineal resection and anal sphincter saving procedures by trying to remain impartial between the desire to perform sphincter preservation and the risk of favoring LR.

## CONCLUSIONS

These premises allow to draw some final consideration, which may be useful for clinical practice. Rectal cancer staging is essential to help clinicians make the right decision on the type of surgery to apply and to determine whether or not neoadjuvant therapy would be appropriate. According to our experience, the local resectability rate for cT3 rectal cancer patients is about 99%. Consequently, TME preceded by RT allows reducing LR rate below 10%. It is therefore essential for surgeons to have a role in multidisciplinary teams in order to have a better control of LR rates and to monitor their own surgical practice quality, as well. It must be stressed that RT or CHT cannot compensate for poor surgery. The dose and fraction of RT we apply seem a good compromise between the long and short course RT, and they are well accepted by our patients. Hopefully, this intermediate RT schedule will have a role in clinical practice in the next future [34]. Surely, it is necessary to introduce further protocols with new CHT treatments able to reduce distant metastases and LR in circumferential resection margin positive patients [35]. The reduction in DR and its impact on overall survival will be the challenge for the future. On the other hand, new prospects, such as the clinical complete response and the “wait-and-see” strategy, should be strengthened, even if, nowadays, this policy is very difficult to follow due to various clinical and ethical reasons [36,37].

## METHODS

### Patients

Patients with cT3 rectal adenocarcinoma located within 10 cm from the anal verge who underwent neoadjuvant RT and with more than 5 years follow-up were included in the analysis. The following exclusion criteria were considered: more than 80 years of age, known metastatic disease and other malignant diseases, and previous RT to the pelvis. A total of 248 consecutive patients who underwent surgery until 2008 were recruited. Preoperative staging was performed by digital rectal examination, colonoscopy and biopsy, abdomino-pelvic computed tomography (CT) and endorectal ultrasound (from 1990), chest x-ray or CT, full blood examination, renal and liver function tests and carcinoembryonic antigen (CEA). The location of the tumor was measured from the anal verge and classified as low rectal (less than 5 cm) or middle rectal (5–10 cm) on the basis of endoscopy and digital rectal examination (cutoff 5 cm). There was no restriction on nodal stage. All the enrolled subjects gave informed written consent to participate to the study protocol, which was approved by the local Scientific and Ethical Committee. The procedures followed were in accordance with the World Medical Association's Declaration of Helsinki (1964, and its later amendments).

### Radiotherapy

RT treatment was performed with a total radiation dose of 36 Gy, delivered in 12 daily fractions of 3 Gy each day for 5 days/week using a 6 or 8 MV x-ray linear accelerator and the four-field box technique; the patient was placed in the prone position. Until June 2002, 146 patients (58.8%) were treated with the 2D conventional technique; thereafter the remaining 102 patients (41.2%) were treated with the 3D conformal technique. The RT treatment plan was established on the basis of the CT scan results. The superior border of the treatment volume was set at the L5-S1 junction; whereas the inferior border was set at the bottom of the ischial tuberosities or at the perineum, depending on the disease extent. The anterior–posterior and posterior–anterior (AP-PA) fields encompassed the whole pelvis with 1.5-2 cm of margin on the bony pelvic inlet; lateral fields encompassed the sacrum posteriorly and the femoral heads anteriorly. For CTV delineation, the AIRO (Associazione Italiana Radioterapia Oncologica) guidelines were used. The three-dimensional conformal RT (3D CRT) with simulation performed on a Toshiba Large Bore CT scanner was performed; the patient was placed in the supine position and his/her legs were immobilized. The same position was maintained for the entire duration of the treatment. The prescribed dose was referred to the axis intersection (ICRU 50).

### Surgery and Histopathological Analysis

Surgery (anterior resection or abdominoperineal excision) was planned before RT and performed within the third week after completion of the RT treatment. It was considered locally curative when, after surgical and histopathological evaluation, the margins of the resected tissue were free of tumor. Sphincter saving (SS) surgery requires a 2-cm distance from the cancer's lower border. Total mesorectal excision (TME) was introduced in 1992. The operative specimen was examined and classified according to the International Union Against Cancer's TNM system [11]. Adjuvant chemotherapy was prescribed according to the referring medical oncologist.

### Follow-up

Each patient was followed-up every 3 months for 2 years, then once every 6 months from the 3^rd^ to the 5^th^ year after surgery; subsequently, once a year. Recurrence located in the pelvic irradiation field was defined as local recurrence (LR); whereas recurrences which were not in this field were considered distant recurrences (DR). Recurrence and survival analysis was limited to patients who underwent curative resection and had a minimum of 5 years of follow-up after surgery at December 2013. The overall survival (OS) was defined as the period of time from the date of surgery until the date of death or the date of the last follow-up for patients who were still alive. Disease-free survival (DFS) was defined as the period of time from the date of surgery until the date of the first local or distant recurrence. Patients who died with no evidence of disease (DOC) were censored at the date of death, and alive patients with no evidence of disease (NED) were censored at the date of the last follow-up. Patients who died with evidence of (local or distant) disease (DOD) were censored at the date of death, and alive patients with evidence of (local or distant) disease (AWD) were censored at the date of the last follow-up.

### Statistical Analysis

Normal distribution assumption was checked by means of Shapiro-Wilks and Kolmogorov-Smirnov tests. Differences in proportions were analyzed using the Chi-square test (Fisher-Yates test). LR and DR and survival analysis were based on all patients who received curative-intent surgery. Survival rates were calculated by using the Kaplan–Meier method for the analysis of censored data, and survival curves were compared with the log rank test. P < 0.05 was considered statistically significant. All analyses were performed and graphs were drawn with SPSS software (IBM SPSS Statistics, IBM Corporation, Chicago, IL).

## List of abbreviations used

AWD: Patients alive with evidence of (local or distant) disease
CHT: chemotherapy
CRT: Chemoradiotherapy
CT: Abdomino-pelvic computed tomography
DFS: Disease free survival
DOC: Patients who died with no evidence of disease
DOD: Patients who died with evidence of disease (local or distal)
DR: Distal recurrence
DS: Sphincter demolition surgery
LR: Local recurrence
MRI: Magnetic resonance imaging
NED: Patients alive with no evidence of disease
OS: Overall survival
OSS-DR: Overall specific survival-DR
OSS-LR: Overall specific survival-LR
pCR: Pathological complete remission
RT: radiotherapy
SD: Standardr deviation
SE: Standard error
SS: Sphincter saving surgery
TME: Total Mesorectal Excision
